# Tumor Suppressor FBXW7 and Its Regulation of DNA Damage Response and Repair

**DOI:** 10.3389/fcell.2021.751574

**Published:** 2021-10-25

**Authors:** Huiyin Lan, Yi Sun

**Affiliations:** ^1^Department of Thoracic Radiation Oncology, Zhejiang Cancer Hospital, Cancer Hospital of University of Chinese Academy of Sciences, Hangzhou, China; ^2^Institute of Cancer and Basic Medicine, Chinese Academy of Sciences, Hangzhou, China; ^3^Cancer Institute of the Second Affiliated Hospital, Institute of Translational Medicine, Zhejiang University School of Medicine, Hangzhou, China; ^4^Cancer Center, Zhejiang University, Hangzhou, China

**Keywords:** cancer, DDR, DNA repair, FBXW7, ubiquitylation

## Abstract

The proper DNA damage response (DDR) and repair are the central molecular mechanisms for the maintenance of cellular homeostasis and genomic integrity. The abnormality in this process is frequently observed in human cancers, and is an important contributing factor to cancer development. FBXW7 is an F-box protein serving as the substrate recognition component of SCF (SKP1-CUL1-F-box protein) E3 ubiquitin ligase. By selectively targeting many oncoproteins for proteasome-mediated degradation, FBXW7 acts as a typical tumor suppressor. Recent studies have demonstrated that FBXW7 also plays critical roles in the process of DDR and repair. In this review, we first briefly introduce the processes of protein ubiquitylation by SCF^FBXW7^ and DDR/repair, then provide an overview of the molecular characteristics of FBXW7. We next discuss how FBXW7 regulates the process of DDR and repair, and its translational implication. Finally, we propose few future perspectives to further elucidate the role of FBXW7 in regulation of a variety of biological processes and tumorigenesis, and to design a number of approaches for FBXW7 reactivation in a subset of human cancers for potential anticancer therapy.

## Introduction

### Protein Ubiquitylation and SCF E3 Ligase With FBXW7 as a Substrate Receptor

Ubiquitylation is a typical post-translational modification, that couples with proteasome, designated as ubiquitin-proteasome system (UPS), as the key proteolytic mechanism in eukaryotes for timely degradation of cellular proteins (Hershko et al., 2000). In general, the UPS-mediated protein degradation includes two steps: (1) covalent attachment of the small peptide ubiquitin to a substrate, a process called ubiquitylation; (2) delivery of ubiquitylated substrates into 26S proteasome for degradation. Ubiquitylation is a well-defined three-step enzymatic cascade catalyzed sequentially by the ubiquitin-activating enzymes (E1s), ubiquitin-conjugating enzymes (E2s), and ubiquitin ligases (E3s) (Ciechanover, 1998). Crucially, E3s determine the substrate specificity through selectively recognizing and directly binding with substrate proteins doomed for ubiquitylation and subsequent degradation.

**FIGURE 1:**
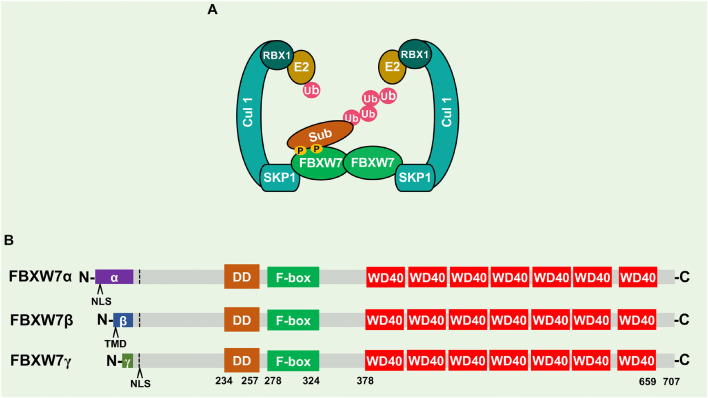
Schematics of SCF, FBXW7, and FBXW7 isoforms. **(A)** SCF^FBXW7^ consists of a scaffold cullin-1 (CUL-1), an adaptor SKP1, a RING-domain protein (RBX1/RBX2), and a substrate-receptor F-box protein (FBXW7). Shown is SCF^FBXW7^ complex in FBXW7 dimerization format for ubiquitylation of a substrate. **(B)** Three FBXW7 isoforms (α, β, and γ) with domain alignment. NLS, nuclear localization signal; TMD, transmembrane domain; DD, dimerization domain.

**FIGURE 2:**
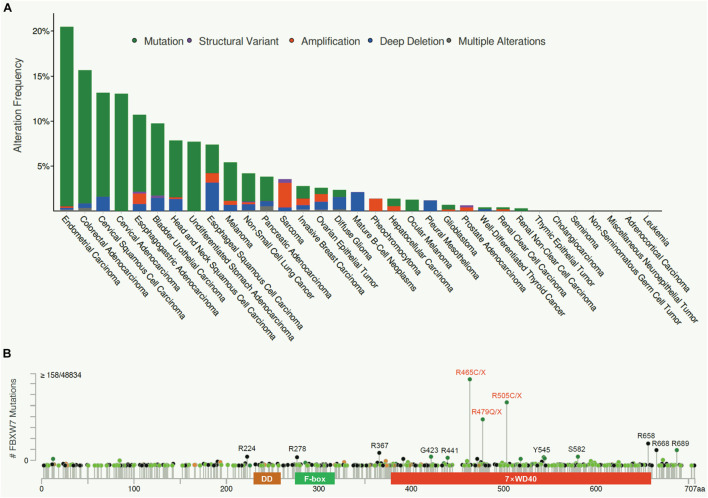
FBXW7 mutation frequency in human cancers and their distributions. **(A)** FBXW7 mutation frequency in different types of human cancer, analyzed from cBioPortal Database (https://www.cbioportal.org/). **(B)** Distribution of FBXW7 mutations in the FBXW7 encoding region. Mutations detected in more than 15 cancer samples are annotated, and three most frequent mutation hotspots, R465, R479, and R505 are highlighted in red.

Among the estimated >600 human E3 ubiquitin ligases, SCF (SKP1-CUL1-F-box protein) is the best studied member of CRL (Cullin-RING-Ligase) family of E3 enzymes. The SCF is a multi-component E3, consisting of a scaffold cullin-1, an adaptor SKP1, a E2 binding RING-domain protein (RBX1/RBX2), and a substrate-receptor F-box protein (Zhao and Sun, 2013; Figure 1A). Although mammalian genome contains 69 F-box proteins (Jin et al., 2004), only three, namely FBXW7, β-TrCP, and SKP2 are well defined and characterized (Skaar et al., 2014). Among these three, FBXW7 is a typical tumor suppressor that promotes the ubiquitylation and degradation of many cellular oncoproteins, and is frequently mutated and inactivated in many human cancers (Welcker and Clurman, 2008; Wang et al., 2012; Figure 2).

### General Introduction of DNA Damage Response and Repair

Mammalian cells are constantly exposed to external and internal insults, such as ionizing radiation (IR), ultraviolet (UV) light, reactive oxygen species (ROS), and many cellular metabolites. These insults typically cause genomic DNA damage in the forms of single strand breaks (SSBs), double strand breaks (DSBs) or replication fork stagnation, and the other types. Among them, DSBs is the most toxic form of DNA damage (Finn et al., 2012).

Upon DSB, a typical DDR is triggered in a cell in an attempt to repair damaged DNA to maintain the genomic integrity. Specifically, three phosphoinositide 3-kinases: ATM (ataxia telangiectasia mutated), ATR (ATM-and RAD3-related), and DNA-PK (DNA-dependent protein kinase) are first recruited into the damage sites and activated via auto-phosphorylation. They then phosphorylate H2AX into γH2AX, which directly binds with MDC1 (mediator of DNA damage checkpoint 1), and recruits the MRN (MRE11-RAD50-NBS1) protein complex to accumulate within the damage sites. The MRN complex acts as an amplifier of DDR signals by enhancing the activity of ATM (Sancar et al., 2004), whereas γ-H2AX is a key mediator for recruitment and retention of high concentration of DNA damage repair enzymes, such as 53BP1, RAD51, and BRCA1 in the vicinity of damaged sites (Nakamura et al., 2010). Following the expansion of DDR signals, two DNA damage repair machineries, namely homologous recombination (HR) and non-homologous end joining (NHEJ), are triggered rapidly and executed to repair the damaged DNA. The failure in DDR and repair is the cause of genomic instability, leading to cell death (if the damage is severe), or various gene mutations to trigger tumorigenesis (Srivastava and Raghavan, 2015).

During the process of DNA damage response and repair, the chromatin and repairing factors are regulated by a spectrum of post-translational modifications including phosphorylation, acetylation, methylation, and ubiquitylation (Wurtele and Verreault, 2006; Van and Santos, 2018). In particular, FBXW7, a receptor protein of SCF E3 ubiquitin ligase, has been recently shown to play fundamental roles in DDR and repair, which is the focus of this review.

## Molecular Characterization of FBXW7

### Isoforms and Subcellular Localizations

The human *FBXW7* gene is localized at chromosome 4q32, a region deleted in 30% of cancers, and encodes three different isoforms (FBXW7α, β, and γ), derived from alternative splicing of the same transcript (Davis et al., 2014). These three isoforms share 10 common exons, encoding three conserved functional domains: (1) the DD dimerization domain, (2) F-box domain to recruit other SCF components, essential for its E3 ligase activity, and (3) substrate recognizing WD40 domain (Hao et al., 2007; Figure 1B). Three isoforms vary at the N-terminus and have different subcellular locations with FBXW7α in the nucleoplasm, FBXW7β in the cytoplasm, and FBXW7γ in the nucleolus (Davis et al., 2014). FBXW7α is functionally the most dominant isoform, which is ubiquitously expressed in most human tissues; FBXW7β expression is mainly found in brain and testis, whereas FBXW7γ is poorly understood and expressed mainly in muscles (Spruck et al., 2002; Matsumoto et al., 2006).

### Dimerization

Dimerization is a common phenomenon and key regulatory modality for FBXW7 (Tang et al., 2007; Welcker and Clurman, 2007; Welcker et al., 2013), as well as for other F-box proteins, such as β-TrCP (Suzuki et al., 2000) and SKP2 (Inuzuka et al., 2012). The FBXW7 dimerization is mediated by the DD domain which enhances its catalytic efficiency for substrate degradation with two possible underlying mechanisms (Tang et al., 2007; Welcker et al., 2013): (1) Dimerization enhances the binding affinity between FBXW7 and substrates. Specifically, the dimer form of SCF^FBXW7^ provides spatial variability for accommodating diverse acceptor lysine geometries in both substrates and ubiquitin chain; and (2) The dimer-orthologs may provide suboptimal and independent recognition sites for substrates, serving as a complementary “buffer” against deleterious mutations in the WD40 domain (Welcker et al., 2013). Furthermore, under overexpressed conditions, FBXW7 could form the stable dimeric form by preventing autoubiquitylation of the monomeric form (Min et al., 2012; Lan et al., 2019). However, a contradictory study showed that while endogenous monomers and dimers are equally stable, the exogenous FBXW7 monomers appears to be more stable than that of dimers (Welcker et al., 2013). Exact reason for this discrepancy is unclear. The authors used wild-type FBXW7 monomer coupled with ubiquitylation-dead FBXW7ΔF monomer and proposed that *trans*-autoubiquitylation may be a major destabilization mechanism. Another possibility is that FBXW7 overexpression may trigger a limiting factor, such as a deubiquitylating enzyme, to block FBXW7 degradation (Welcker et al., 2013).

Recently, we and the others found that FBXW7 dimerization was regulated by several FBXW7-interacting proteins. For example, the prolyl isomerase PIN1 interacts with FBXW7 to prevent its dimerization in a phosphorylation-dependent manner (Min et al., 2012), whereas LSD1 directly binds to FBXW7 to disrupt FBXW7 dimerization, leading to its self-ubiquitylation (Lan et al., 2019).

### Phosphorylation

FBXW7 was also regulated by phosphorylation which also affects its stability. One study showed that extracellular signal-regulated kinase (ERK) directly interacted with and phosphorylated FBXW7 at Thr^205^, which promoted FBXW7 ubiquitylation in a PIN-1 dependent manner in pancreatic cancer cells, although exactly how ERK-mediated FBXW7 phosphorylation triggers FBXW7 ubiquitylation remains elusive (Ji et al., 2015). Nevertheless, the study revealed a new mechanism by which the Kras-MAPK signal promotes pancreatic tumorigenesis via promoting degradation of tumor suppressor FBXW7. Another study showed that PLK1 phosphorylates FBXW7 at Ser^58^ and Thr^284^ to promote FBXW7 self-ubiquitylation, leading to stabilization of FBXW7 substrate N-MYC, which in turn transactivates PLK1, thus establishing a positive feed-forward loop that enhance MYC-regulated oncogenic programs (Xiao et al., 2016). On the contrary, two studies showed that FBXW7 phosphorylation at Ser^227^ by either serum and glucocorticoid-regulated kinase 1 (SGK1) or phosphoinositide 3-kinase (PI3K), respectively, switched the catalytic activity of FBXW7 toward its substrates instead of targeting itself for self-ubiquitylation (Mo et al., 2011; Schulein et al., 2011). Thus, FBXW7 phosphorylation appears to play critical role in FBXW7 stability in the manner dependent of the kinases and their phosphorylation sites. In addition, protein kinase (PK) C mediated FBXW7 phosphorylation at Ser^8^/Ser^10^ was reported to be involved in the nuclear localization of FBXW7α (Durgan and Parker, 2010).

### Mutations in Human Cancers

Consistent with its role as a tumor suppressor, FBXW7 is the most frequently mutated gene among all the genes encoding F-box proteins in human cancers. We performed the meta-analyses of the cBioPortal Database^1^ and found an overall FBXW7 somatic mutation rate of 3.23% in human cancers (1,497 cases out of 46,305 tested), though different cancer types exhibit different mutational spectra (Figure 2A). The cancer with the highest mutational frequency is endometrial cancer (20.5%), followed by colorectal cancer (15.7%), cervical cancer (13.6), and esophagogastric adenocarcinoma (10.7%). Significantly, most of the FBXW7 mutations are single nucleotide change, resulting in single amino acid substitutions, within the WD40 domains responsible for substrate binding. As such, the mutations of these key residues often disrupt FBXW7 binding with its oncogenic substrates. Figure 2B showed three mutation hotspots R465, R479, and R505 (Welcker and Clurman, 2008; Davis et al., 2014), representing as much as 27.3% of cases (408/1497) found in all FBXW7 mutations, along with other mutation sites detected in more than 15 cases in all human cancers, including R224, R278, R367, G423, R441, Y545, S582, R658, R668, and R689.

### Known FBXW7 Substrates

FBXW7 is well-known for its tumor suppressor function against cancer development by targeting a variety of oncoproteins for proteasomal degradation. A majority of FBXW7 substrates identified and characterized to date were summarized in Table 1. Almost all substrates contain a evolutionarily conserved phosphorylation motif, designated as CDC4 phospho-degron (CPD), of which substrate phosphorylation is a prerequisite event for FBXW7 binding and subsequent ubiquitylation (Nash et al., 2001). Among all FBXW7 substrates, most of them are well-known oncoproteins, which play the key roles in regulation of cell growth, apoptosis, differentiation and cell migration among the others (Wang et al., 2012). Some of these oncogenic substrates, such as c-MYC, NOTCH 1, MCL-1, Cyclin E, and c-JUN, likely play a driver role in FBXW7-associated cancers (Fryer et al., 2004; Wei et al., 2005; Welcker and Clurman, 2008; Davis et al., 2014). Taking c-MYC as an example, the studies using both *in vitro* and *in vivo* models showed that c-MYC is a classic substrate of FBXW7. Specifically, FBXW7-mediated c-MYC degradation relies on prior CPD phosphorylation of C-MYC at Thr^58^ and Ser^62^ by GSK3 and MAPK, respectively (Welcker et al., 2004; Yada et al., 2004). It is, therefore, not surprising that the point mutations on Thr^58^ and Ser^62^ were found on *c-Myc* in a variety of human cancers, thus avoiding FBXW7 degradation and being selected with growth advantage (Bahram et al., 2000). Moreover, in several *fbxw7* KO mouse models, c-Myc was remarkably accumulated to accelerate tumorigenesis and promote tumor growth (Yada et al., 2004; Onoyama et al., 2007).

**TABLE 1:** Summary of FBXW7 substrates.

Substrates	Functions/Pathways	Kinase(s)	References
Aurora A	Protein kinase	GSK3	Kwon et al., 2012
Aurora B	Protein kinase	–	Teng et al., 2012
BLM	DNA helicase	GSK3, CDK2	Kharat et al., 2016
B-Raf	Protein kinase	ERK	de la Cova and Greenwald, 2012
Brg1	Transcription factor	CK1	Huang et al., 2018
C/EBPα	Transcription factor	–	Bengoechea-Alonso and Ericsson, 2010a
C/EBPδ	Transcription factor	GSK3	Balamurugan et al., 2013
CCDC6	ATM substrate	–	Zhao et al., 2012
c-JUN	Transcription factor	GSK3	Nateri et al., 2004; Wei et al., 2005
c-MYB	Transcription factor	GSK3, NLK	Kanei-Ishii et al., 2008; Kitagawa et al., 2009
c-MYC	Transcription factor	GSK3, MAPK	Welcker et al., 2004; Yada et al., 2004
CREB3L1/2	Transcription factor	–	Yumimoto et al., 2013
Cyclin E	Cyclin protein, Cell cycle	CDK2, GSK3	Koepp et al., 2001; Moberg et al., 2001; Strohmaier et al., 2001
DEK	Chromatin regulator	GSK3	Babaei-Jadidi et al., 2011
ERG	TMPRSS2-ERG fusion protein	GSK3, WEE1	Hong et al., 2020
FAAP20	Subunit of FA core complex	GSK3	Wang et al., 2016
Fetuin-A	Alpha-2-HS-glycoprotein	–	Zhao et al., 2018
GCSF-R	Cytokine receptor	GSK3	Lochab et al., 2013
GRα	Transcription factor	GSK3	Malyukova et al., 2013
HIF1α	Transcription factor	GSK3	Cassavaugh et al., 2011
Jun B	Transcription factor	GSK3	Perez-Benavente et al., 2011
KLF13	Transcription factor	GSK3	Kim et al., 2012
KLF2	Transcription factor	GSK3	Wang et al., 2013
KLF5	Transcription factor	GSK3	Zhao et al., 2010
MCL-1	Bcl2 family protein	GSK3	Inuzuka et al., 2011b; Wertz et al., 2011
MED13/13L	Component of mediator complex	–	Davis et al., 2013
mTOR	Protein kinase	–	Mao et al., 2008
NF1	Ras GTPase regulator	–	Tan et al., 2011
Notch1	Transcription factor	CDK8	Fryer et al., 2004
Nrf1	Transcription factor	GSK3	Biswas et al., 2011
p100	Transcription factor	GSK3	Arabi et al., 2012; Busino et al., 2012; Fukushima et al., 2012
p53	Transcription factor	GSK3, ATM	Galindo-Moreno et al., 2019; Tripathi et al., 2019; Cui et al., 2020
p63	Transcription factor	GSK3	Galli et al., 2010
PDC-1α	Nuclear receptor co-activator	GSK3, p38	Olson et al., 2008
PLK1	Serine/threonine kinase	GSK3	Giraldez et al., 2014
Presenilin	Protease	–	Wu et al., 1998
PU.1	Transcription factor	GSK3	Mishra et al., 2021
RCAN1	Calcineurin A binding protein	–	Lee et al., 2012
REV-ERBα	Nuclear receptor	CDK1	Zhao et al., 2016
RIG-1	RNA helicase	–	Wang et al., 2017
SHOC2	RAS activator	MAPK	Xie et al., 2019
SOX9	Transcription factor	GSK3	Hong et al., 2016; Suryo Rahmanto et al., 2016
SRC-3	Nuclear receptor co-activator	GSK3	Wu et al., 2007
SREBP1	Transcription factor	GSK3	Sundqvist et al., 2005
TG1F1	Transcription factor	–	Bengoechea-Alonso and Ericsson, 2010b
Topo IIα	Topoisomerase	GSK3, CK2	Chen et al., 2011
TPP1	Telomere protection protein 1	GSK3	Wang et al., 2020
XRCC4	DNA repair protein	DNA-PKcs	Zhang et al., 2016
ZNF322A	Transcription factor	CK1, GSK3	Liao et al., 2017
γ-Catenin	Transcription factor	–	Li et al., 2018
ΔNp63α	Transcription factor	GSK3	Galli et al., 2010

In addition to these classical substrates targeted by FBXW7 for ubiquitylation and degradation, several non-canonical substrates were also targeted by FBXW7, but not for degradation (Lan and Sun, 2019). For instance, FBXW7 mediated K63-linked polyubiquitylation of XRCC4 to facilitate the NHEJ repair (Zhang et al., 2016); whereas polyubiquitylation of γ-catenin via K63-linkage by FBXW7 led to enhanced suppression of cell proliferation and G2/M cell cycle transition (Li et al., 2018). Most recently, we found that LSD1 acts as a FBXW7 pseudo-substrate, not being ubiquitylated by FBXW7, but triggering FBXW7 self-ubiquitylation and degradation via disrupting FBXW7 dimerization (Lan et al., 2019). Likewise, EBNA1-binding protein 2 (Ebp2) was also shown to behave as a pseudo-substrate, which directly binds with FBXW7γ in the canonical CPD-dependent manner, not for its degradation, but facilitating the nucleolar localization of FBXW7γ (Welcker et al., 2011).

## The Role of FBXW7 in DNA Damage Response

By targeting a wide array of protein substrates for ubiquitylation and degradation, FBXW7 was involved in regulatory networks of various biological processes such as cell cycle progression, cell differentiation, apoptosis, and autophagy (Welcker and Clurman, 2008; Davis et al., 2014). However, knowledge on whether and how FBXW7 regulates DNA damage response (DDR) and repair is rather limited. An early study showed that genetic inactivation of *hCDC4* (encoding FBXW7) in karyotype stable colorectal cancer cells resulted in nuclear atypia, such as micronuclei and lobulated or elongated nuclei, as well as chromosomal instability, as evidenced by increased multipolar spindles and euploidy in a manner dependent of cyclin E accumulation (Rajagopalan et al., 2004). However, no rescue experiment nor detailed underlying mechanism was provided to demonstrate whether cyclin E is indeed playing a causal role. Nevertheless, this study did implicate that FBXW7 is likely involved in the maintenance of the genomic integrity. Another *in vivo* study showed that *Fbxw7* is a p53-dependent haploinsufficiency tumor suppressor, and *Fbxw7*^±^ mice are much more susceptible to radiation-induced tumorigenesis (Mao et al., 2004). A recent study also showed that *Fbxw7*^±^ mice have an increased the risk of developing gastric cancer induced by chemical carcinogen N-methyl-N-nitrosourea (MNU), which is dependent of the accumulation of DNA damage and c-Myc oncoprotein (Jiang et al., 2017).

**FIGURE 3:**
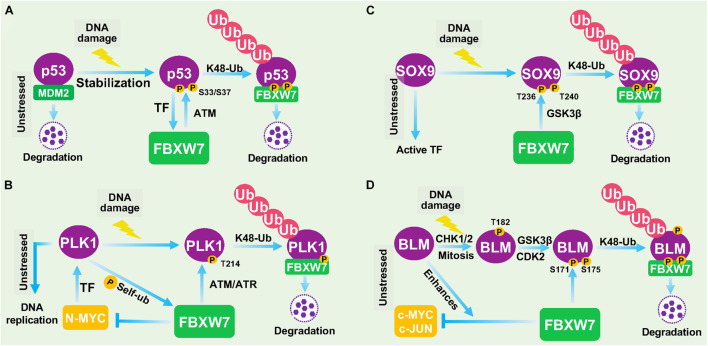
FBXW7 in DNA damage responses. **(A)** Negative feedback loop between FBXW7 and p53. Under unstressed condition, p53 level is low due to targeted degradation by MDM2 E3 ligase. Upon DNA damage, p53 protein is accumulated to transcriptionally induce FBXW7 expression; the induced FBXW7 then promotes p53 ubiquitylation and degradation after ATM-mediated p53 phosphorylation at Ser^33/37^ residues (Cui et al., 2020). **(B)** PLK1 degradation by FBXW7 upon DDR. Under unstressed condition, PLK1 was transcriptionally induced by N-MYC. PLK also phosphorylates FBXW7 to trigger its self-ubiquitylation and cause N-MYC accumulation. Upon DNA damage, ATM/ATR phosphorylates PLK1 at Thr^214^, which facilitated FBXW7 binding and subsequent ubiquitylation and degradation. **(C)** FBXW7 degrades SOX9 upon DDR. Under unstressed condition, SOX9 acts as a transcription factor. Upon DNA damage, SOX9 was phosphorylated at Thr^236/240^ by GSK3β, which facilitated FBXW7 binding and subsequent ubiquitylation and degradation. **(D)** FBXW7 degrades BLM upon DDR. Upon DNA damage, BLM was sequentially phosphorylated at Thr^182^ by CHK1/2, and at Thr^171^ and Ser^175^ by GSK3β/CDK2, which facilitated FBXW7 binding and subsequent ubiquitylation and degradation. TF, Transcription factor.

Recently, more studies showed that FBXW7 is indeed involved in DDR. Upon DNA damage, FBXW7 was found to promote the ubiquitylation and degradation of several key DDR regulatory proteins, including p53 (Galindo-Moreno et al., 2019; Tripathi et al., 2019; Cui et al., 2020), polo-like kinase 1 (PLK1; Strebhardt, 2010), SOX9 (Hong et al., 2016), and bloom (BLM) helicase (Kharat et al., 2016) among others, which is described below (Figures 3A–D).

### Negative Feedback Loop Between FBXW7 and p53

p53 is the best-known tumor suppressor in human cancer, acting as a transcription factor to regulate a wide range of cellular processes, including growth arrest, apoptosis, senescence, DDR, and DNA repair (Vogelstein et al., 2000). The abundance and activity of p53 were fine-tuned by multiple cellular signals and post-translational modifications. In response to DNA damage and other cellular stresses, p53 level is usually increased due to enhanced protein stabilization, mainly resulting from disruption of Mdm2 binding and Mdm2-mediated degradation (Wade et al., 2013). An early study reported that FBXW7 expression was transcriptionally induced upon p53 accumulation after DNA damage stress, thus demonstrating FBXW7 as a *bona fide* transcriptional target of p53 using cell culture models (Kimura et al., 2003). A subsequent study using a mouse model confirmed that *Fbxw7* is indeed a p53-dependent, haploinsufficient tumor suppressor gene (Mao et al., 2004). Very interestingly, three recent studies showed that p53 is also subject to post-translational regulation by FBXW7 for targeted degradation in response to DNA damage in multiple human cancer cell lines (Galindo-Moreno et al., 2019; Tripathi et al., 2019; Cui et al., 2020), thus demonstrating FBXW7 participation in DDR to protect cancer cells from DNA damage-induced cell cycle arrest and apoptosis. Collectively, it is apparent that a negative feedback loop exists between FBXW7 and p53. In response to DNA damage, p53 protein is accumulated to transcriptionally induce FBXW7 expression; the induced FBXW7 then promotes p53 degradation to keep the p53 level in check as a mechanism of self-defense (Figure 3A). Given both FBXW7 and p53 are frequently mutated in many types of human cancers, we performed a bioinformatics analysis on TCGA databases and found interestingly that mutations of p53 and FBXW7 in human cancers are co-occurrence (Zehir et al., 2017), suggesting that this p53-FBXW7 negative feedback loop may have a biological implication.

### FBXW7 Degrades PLK1 in Response to DNA Damage

PLK1 is a serine/threonine-protein kinase that performs important biological functions mainly in the late G2/M phase of cell cycle, including centrosome maturation, spindle assembly and sister chromatid separation (Strebhardt, 2010). PLK1 was also shown to promote DNA replication by regulating pre-replicative complexes (pre-RCs) loading of mini-chromosome maintenance (MCM) 2/6 (Yoo et al., 2004; Tsvetkov and Stern, 2005). In response to UV-induced DNA damage, PLK1 was degraded by FBXW7 in cells arrested at G1- and S-phase, thus blocking the formation of pre-RCs to prevent the improper progression of cell cycle and avoid the proliferation of cells carrying damaged DNA (Giraldez et al., 2014). Thus, by degrading PLK1 to temporarily halt cell cycle progression, FBXW7 acts as a gate-keeper to ensure genome stability (Giraldez et al., 2014; Figure 3B).

### FBXW7 Degrades SOX9 in Response to DNA Damage

SOX9 is a member of the high-mobility group (HMG)-box class of transcription factors, and plays a key role in chondrocytes differentiation and skeletal development (Adam et al., 2015). It was reported that under the DNA damage induced by UV irradiation or genotoxic chemotherapeutic agents, SOX9 was actively degraded in various cancer types and even in normal epithelial cells. Mechanistic study revealed that FBXW7 is the E3 ubiquitin ligase mediating SOX9 degradation in a manner dependent on prior phosphorylation by GSK3β (Hong et al., 2016; Figure 3C). However, how FBXW7-mediated SOX9 degradation contributes to overall DDR remains elusive. Furthermore, SOX9 protein was also targeted by FBXW7 for proteasomal degradation in medulloblastoma cells even under normal unstressed conditions (Suryo Rahmanto et al., 2016), suggesting that FBXW7-mediated SOX9 degradation might be in a cell and context dependent event, and not specific to DDR.

### FBXW7 Degrades Bloom Helicase in Response to DNA Damage

BLM is an ATP-dependent DNA helicase that unwinds single- and double-stranded DNA. Once stalled DNA replication or DNA damage occurs, BLM is recruited to participate in fixing the genomic error (Chu and Hickson, 2009). The interaction between BLM and FBXW7 has been previously reported to enhance FBXW7-mediated c-MYC degradation (Chandra et al., 2013). A more recent study showed that the protein levels of BLM is dynamically fluctuant during cell cycle progression with the interphase cells having a higher level than the mitotic cells. Further mechanistic studies revealed that FBXW7 promoted the K48-linked polyubiquitylation of BLM in a manner dependent on GSK3β and CDK2 -mediated prior phosphorylation of BLM at Thr^171^ and Ser^175^ (Kharat et al., 2016). The authors further found that FBXW7-promoted BLM degradation is a mitosis-specific event requiring prior phosphorylation at Thr^182^ by CHK1/CHK2 (Figure 3D). Given that CHK1/CHK2 activation is a common signal during DDR, FBXW7-mediated BLM degradation triggered by CHK1/CHK2 is likely involved in the process of DDR, although detailed underlying mechanism remains elusive.

Furthermore, we recently found that FBXW7 was recruited to the DSB sites by poly(ADP-ribose) (PAR) (Zhang Q. et al., 2019) and maintained at DNA damage sites in a ATM-dependent manner upon laser irradiation to facilitate the non-homolog end-joining (NHEJ) repair (Zhang et al., 2016) (see below). The others reported that FBXW7 binds to telomere protection protein 1 (TPP1) and promotes its polyubiquitylation at multi-sites for enhanced degradation, which triggers telomere uncapping and DNA damage response and affects senescence and fibrosis of pulmonary epithelial stem cell (Wang et al., 2020). Upon DNA damage stress in prostate cancer (PCa) cells, *TMPRSS2-ERG* gene fusion product was degraded by FBXW7 in a manner dependent of GSK3β and WEE1 kinases. Blockage of such degradation promoted genotoxic therapy-resistant growth of fusion-positive PCa cells both *in vitro* and *in vivo* (Hong et al., 2020). In nasopharyngeal carcinoma cells, fructose-1,6-bisphosphatase 1 (FBP1) was found to suppress the autoubiquitylation of FBXW7 to restrained mTOR-glycolysis signals and promote radiation-induced apoptosis and DNA damage (Zhang et al., 2021). FBXW7 also cooperated with MDM2 following DDR, to regulate the levels of the pro-proliferative ΔNp63α protein, resulting in cell proliferation (Galli et al., 2010). Finally, in addition to p53, other transcription factors known to be FBXW7 substrates were also reported to be involved in DDR. For example, HIF-1 was involved in γH2AX accumulation by tumor hypoxia (Wrann et al., 2013), whereas KLF5 plays a significant role in the DNA damage response by regulating the phosphorylation of CHK1/2 (Zhang H. et al., 2019). Taken together, it appears that FBXW7 is indeed implicated in DDR that likely contribute to its role in the maintenance of genome integrity, mainly by targeted ubiquitylation and degradation of key regulatory proteins, such as p53, PLK1, and BLM.

## The Role of FBXW7 in DNA Damage Repair

**FIGURE 4:**
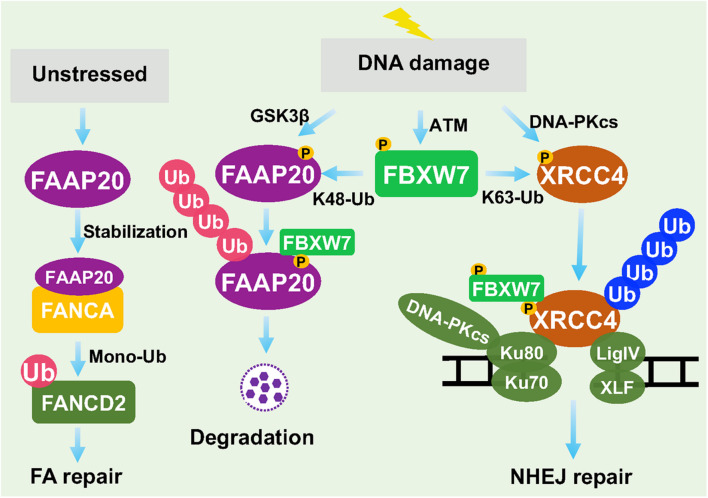
FBXW7 in regulation of DNA repair. Under unstressed condition, FAAP20 binds FANCA to facilitate mono-ubiquitylation of FANCD2. Upon DDR, FAAP20 was phosphorylated by GSK3β to facilitate FBXW7 binding and ubiquitylation. On the other hand, DNA-PKcs phosphorylated XRCC4, whereas ATM phosphorylated FBXW7. At the damage site, FBXW7 promoted XRCC4 polyubiquitylation via the K63 linkage to facilitate recruitment of Ku70/Ku80 for NHEJ repair.

The DDR and repairs are two sequential events which are essential for the maintenance of genomic stability. The DNA attack by different external and internal insults produces a variety of DNA lesion modalities, mainly including simple base modification, base mismatches, bulky DNA adducts, inter-strand and intra-strand crosslinks (ICLs), SSBs and DSBs, which trigger different types of DDRs and repair processes to fix these damages (Roos et al., 2016). For example, base mismatches are repaired by mismatch repair machinery (MMR), ICLs are repaired by NER, HR, and Fanconi Anaemia (FA) repair pathways, whereas DSBs are mainly repaired by NHEJ or HR (Roos et al., 2016). So far, FBXW7 was found to regulate FA and NHEJ pathways by targeting Fanconi anemia core complex-associated protein (FAAP) 20 (Wang et al., 2016, 2019) and XRCC4 (Zhang et al., 2016), respectively (Figure 4).

### In Regulation of Fanconi Anemia Pathway

The Fanconi Anemia (FA) pathway is a DNA repair process responsible for resolving ICLs (Roos et al., 2016). Germ-line mutations of key FA genes caused inherited FA disorders with cancer predisposition. The key step of FA pathway to initiate the repair process is the monoubiquitylation of FANCD2, mediated by FA core complex containing a scaffold protein FANCA. FANCA has a binding partner, FAAP20, which is required for its stability (Leung et al., 2012). It was reported that FBXW7 promoted the polyubiquitylation of FAAP20 in a manner dependent on GSK3β-mediated prior phosphorylation at Ser^113^ in response to DNA damage. Thus, by modulating the levels of FAAP20 and subsequent stability of FANCA, FBXW7 acts as a upstream regulator of FA pathway and its repair process (Wang et al., 2016).

### In Regulation of Non-homologous End Joining Repair

Recently, we reported that in response to DSB DNA damage, ATM is activated to remain FBXW7 at the damage sites and then trigger NHEJ, but not HR, repair (Zhang et al., 2016). Specifically, ionizing radiation caused DNA damage that activates both DNA-PKcs and ATM. On the one hand, DNA-PKcs phosphorylates XRCC4, a key regulator of NHEJ on damaged site, and on the other hand, PAR and ATM recruit FBXW7 to the DSBs sites, where FBXW7 promotes polyubiquitylation of phosphorylated XRCC4 via the K63 linkage. Polyubiquitylated XRCC4 was not delivered to proteasome for degradation, rather to build up a platform that facilitated the recruitment of Ku70/80 heterodimer to promote NHEJ repair (Zhang et al., 2016; Zhang Q. et al., 2019). Thus, FBXW7 is actively involved in processes of DNA damage repair by FA and NHEJ.

Furthermore, it is worth noting that c-MYC, as a typical substrate of FBXW7, was also reported to function in DNA damage repair. Specifically, it was reported that c-MYC suppresses DSB accumulation in a manner strictly dependent of Polymerase Associated Factor 1 complex (Endres et al., 2021). Another study reported that c-MYC directly interacts with Ku70 protein through its Myc box II (MBII) domain to block DSB repair and V(D)J recombination, which probably occur through inhibition of the NHEJ pathway (Li et al., 2012). Given the fact that FBXW7 has a variety of substrates, it is likely that some of these substrates may regulate DNA damage repair in an indirect manner, if not in a direct manner, which is, however, out of scope of this focused review.

## Conclusion and Future Perspectives

In the past two decades, the FBXW7-related studies were mainly focused on identification and characterization of its substrates. To date, more than 40 proteins have been identified, and most of them are transcription factors that regulate a broad range of biological processes. By timely modulating ubiquitylation and degradation of these substrates, FBXW7 acts as a central regulator of key biological processes and various cellular signal pathways. However, the knowledge on the role of FBXW7 in DDR and repair is rather limited, though the involvement of FBXW7 in genomic stability was implicated almost two decades ago. In this review, we summarized current available data and concluded that FBXW7 is indeed actively involved in DDR and repair processes by acting as an E3 ligase to promote ubiquitylation of (a) key DDR regulatory proteins for degradation or (b) key repair proteins for facilitating repair process (Figures 3, 4).

**FIGURE 5:**
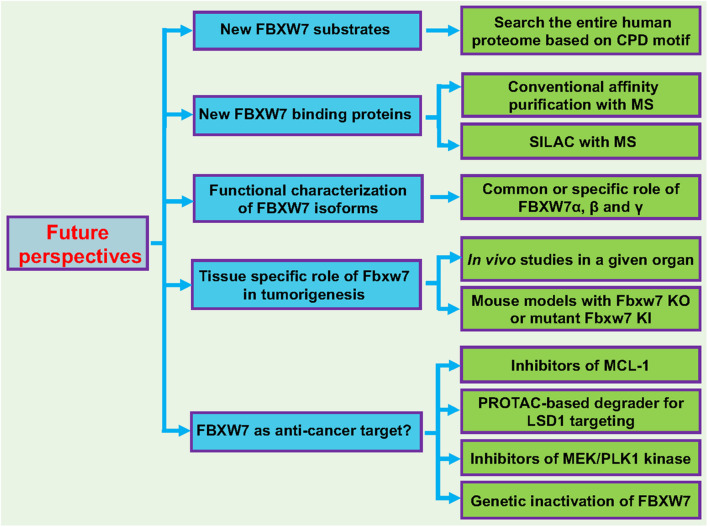
Future perspectives. Five future directions are proposed to further broaden our understanding of FBXW7 functions: (1) Identification and characterization of new FBXW7 substrates; (2) Identification and characterization of FBXW7 binding proteins; (3) Functional characterization of FBXW7 isoforms (α, β, and γ); (4) Tissue specific role of Fbxw7 in tumorigenesis using mouse cKO or KI models; (5) Target human cancers by reactivating FBXW7. See text for details.

Here, we propose few future studies on FBXW7 to further elucidate the role of FBXW7 in a variety of biological processes (Figure 5).

### Identification and Characterization of Additional FBXW7 Substrates Which May or May Not Be Involved in DNA Damage Response and Repair Processes

Given that FBXW7 binding motif, also known as CDC4 phospho-degron (CPD), on a substrate has been well-defined, the quick and easy way to identify new putative FBXW7 substrate candidates is to search the entire human proteome for CPD motif or its modified version with mimicking negative charged residues within the CPD motif, followed by detailed characterization of candidates as the *bona fide* FBXW7 substrates. FBXW7 functions will be further extended, based upon the known functions of these newly identified substrates in a given signaling pathway.

### Identification and Characterization of FBXW7 Binding Proteins That Regulate FBXW7 Functions

Conventional affinity purification coupled with Mass-Spectrometry (MS) or stable isotope labeling by amino acids in cell culture (SILAC) with the MS-based quantitative proteomics (Ong and Mann, 2006) can be used to identify FBXW7 binding proteins under unstressed physiological condition or after a given stress of interest. The identified binding proteins can also be FBXW7 substrates or merely FBXW7 binding proteins not subjected to FBXW-mediated ubiquitylation. FBXW7 may have novel functions independent of its E3 ligase activity, which can be identified and defined through the characterization of these binding partners.

### Functional Characterization of FBXW7 Isoforms

The majority of current studies have been focused upon FBXW7α localized in the nucleus. No detailed functional characterization of other isoforms, particularly FBXW7γ localized in the nucleolus. It is unclear whether other SCF components are localized in nucleolus and if not, FBXW7γ may not act as an active E3 ligase. It will be interesting to define the role of this isoform, particularly in response to ribosomal stress that is mainly triggered in nucleolus (Golomb et al., 2014), a subcellular organelle which also plays a role in maintenance of genome stability (Lindstrom et al., 2018).

### Tissue Specific Role of Fbxw7 in Tumorigenesis

In human cancer cells, FBXW7 is frequently inactivated via point mutations, allele deletion, promoter methylation, and induced self-ubiquitylation (Welcker and Clurman, 2008; Wang et al., 2012; Lan and Sun, 2019). It is very likely that cancer cells with FBXW7 inactivation have growth advantage and were selected during tumorigenesis. However, these correlation-based studies did not validate whether FBXW7 truly plays a causal role in organ-specific tumorigenesis. Thus, for *in vivo* validation of the role of FBXW7 in tumorigenesis in a given organ, the mouse models with tissue-specific *Fbxw7* KO or mutant *Fbxw7* KI should be generated, particularly in those tissues with high frequency of FBXW7 alterations (Figure 2A). A detailed characterization of these genetically modified mouse models will reveal whether FBXW7 indeed plays a driver role or merely co-operate with other dominant oncogenes (such as Kras activation) or tumor suppressors (such as loss of p53 or Pten) in compound mouse models in a particular organ.

### FBXW7 as a Potential Anti-cancer Target?

FBXW7 is a tumor suppressor and FBXW7 itself certainly cannot be served as a direct anti-cancer target. However, few correlation studies have shown an association of FBXW7 expression with chemotherapeutic sensitivity. For example, loss of FBXW7 was associated with increased sensitivity of lung cancer cells to histone deacetylase (HDAC) inhibitor, MS-275 (Yokobori et al., 2014), and tumor cell lines harboring deletions or mutations in FBXW7 are particularly sensitive to rapamycin treatment (Mao et al., 2008). Given the fact that pro-survival protein MCL-1 is a substrate of FBXW7, it is not surprised that T-cell acute lymphoblastic leukemia cell lines with defective FBXW7 have increased levels of MCL1 and are particularly resistance to BCL2 antagonist ABT-737 in a manner dependent of MCL1 (Inuzuka et al., 2011a). Furthermore, FBXW7 loss conferred resistance to anti-tubulin agents and promoted chemotherapeutic-induced polyploidy due to MCL1 accumulation (Wertz et al., 2011). These studies together suggest that loss-of-function FBXW7 mutations indeed impact chemotherapy sensitivity, although it is mainly due to accumulation of its anti-apoptotic substrates, not directly related to its DNA repair function. Nevertheless, small molecule inhibitors of MCL1, currently under clinical development (Hird and Tron, 2019; Negi and Murphy, 2021), are certainly a proper choice to target human cancers with FBXW7 mutations.

On the other hand, the upstream regulators of FBXW7 also provide sound strategies for potential FBXW7-based translational application in cancer treatment. Specifically, several oncoproteins have been shown to trigger FBXW7 self-ubiquitylation and targeting these oncoproteins would, in theory, stabilize FBXW7 to execute its tumor suppressor function.

The first case is LSD1 (lysine-specific demethylase-1), an enzyme overexpressed in many human cancers with correlation of poor patient survival (Kooistra and Helin, 2012), which has been validated as an attractive cancer target with extensive drug discovery efforts (Fu et al., 2017). We recently found that LSD1 is a pseudo FBXW7 substrate, which binds with FBXW7 in the classical CPD-dependent manner. Instead of being ubiquitylated by FBXW7 for proteasomal degradation, LSD1-FBXW7 binding inhibited FBXW7 dimerization, leading to FBXW7 self-ubiquitylation and subsequent degradation via proteasome and lysosome systems in a manner independent of its demethylase activity (Lan et al., 2019; Gu et al., 2020). Currently, several LSD1 demethylase inhibitors have been in the Phase I/IIa clinical trials (Hosseini and Minucci, 2017). Future drug discovery effort could be directed to screen for small molecules that specifically bind to LSD1, not necessary to inhibit its demethylase activity, followed by discovery of PROTAC-based degrader (Gu et al., 2018) for LSD1 targeting. This type of LSD1 specific PROTAC drugs should have broad applications for the treatment of human cancers harboring a wild type FBXW7 with LSD1 overexpression by reactivating FBXW7, as well as for potential immunotherapy in combination with PD-L1 blockade (Sheng et al., 2018).

The second example is the ERK kinase that has been shown to phosphorylate FBXW7, leading to its destabilization in a manner dependent of PIN-1 (Ji et al., 2015). The inhibitors of MEK, an ERK upstream kinase, have also been in a number of Phase II/III clinical trials (Kim and Giaccone, 2018). The third case is the PLK kinase that has also been shown to phosphorylate FBXW7 to promote its self-ubiquitylation (Xiao et al., 2016). Again, several PLK inhibitors were also under clinic development (Janning and Fiedler, 2014). These inhibitors may have new applications in the treatment of human cancers with activated MAPK or PLK1 signals by reactivating FBXW7. Finally, in a subset of human cancers with developed resistance to chemo- and radiotherapies, but harboring wild-type p53, inactivation of FBXW7 via genetic approaches may reactivate p53 to overcome the resistance (Cui et al., 2020).

## Author Contributions

HL wrote the first draft of the manuscript. YS revised and finalized the manuscript. Both authors contributed to the article and approved the submitted version.

## Conflict of Interest

The authors declare that the research was conducted in the absence of any commercial or financial relationships that could be construed as a potential conflict of interest.

## Publisher’s Note

All claims expressed in this article are solely those of the authors and do not necessarily represent those of their affiliated organizations, or those of the publisher, the editors and the reviewers. Any product that may be evaluated in this article, or claim that may be made by its manufacturer, is not guaranteed or endorsed by the publisher.

## Footnotes

1
https://www.cbioportal.org/
